# Physical Activity, Physical Performance, and Biological Markers of Health among Sedentary Older Latinos

**DOI:** 10.1155/2014/535071

**Published:** 2014-06-12

**Authors:** Gerardo Moreno, Carol M. Mangione, Pin-Chieh Wang, Laura Trejo, Anthony Butch, Chi-Hong Tseng, Catherine A. Sarkisian

**Affiliations:** ^1^UCLA Department of Family Medicine, 10880 Wilshire Boulevard, Suite 1800, Los Angeles, CA 90024, USA; ^2^UCLA Department of Medicine, Division of General Internal Medicine & Health Services, 911 Broxton Avenue, Los Angeles, CA 90095, USA; ^3^UCLA Department of Medicine, Division of Geriatrics, 10945 Le Conte Avenue, Suite 2339, Los Angeles, CA 90095, USA; ^4^City of Los Angeles Department of Aging, 221 North Figueroa Street, Suite 180, Los Angeles, CA 90012, USA; ^5^UCLA Department of Pathology & Laboratory Medicine, 10833 Le Conte Avenue, Los Angeles, CA 90095, USA; ^6^VA Greater LA Healthcare System, Building 220, 11301 Wilshire Boulevard, Los Angeles, CA 90073, USA

## Abstract

*Background*. Physical activity is associated with better physical health, possibly by changing biological markers of health such as waist circumference and inflammation, but these relationships are unclear and even less understood among older Latinos—a group with high rates of sedentary lifestyle. *Methods*. Participants were 120 sedentary older Latino adults from senior centers. Community-partnered research methods were used to recruit participants. Inflammatory (C-reactive protein) and metabolic markers of health (waist circumference, HDL-cholesterol, triglycerides, insulin, and glucose), physical activity (Yale physical activity survey), and physical performance (short physical performance NIA battery) were measured at baseline and 6-month followup. *Results*. Eighty percent of the sample was female. In final adjusted cross-sectional models, better physical activity indices were associated with faster gait speed (*P* < 0.05). In adjusted longitudinal analyses, change in self-reported physical activity level correlated inversely with change in CRP (*β* = −0.05; *P* = 0.03) and change in waist circumference (*β* = −0.16; *P* = 0.02). Biological markers of health did not mediate the relationship between physical activity and physical performance. *Conclusion*. In this community-partnered study, higher physical activity was associated with better physical performance in cross-sectional analyses. In longitudinal analysis, increased physical activity was associated with improvements in some metabolic and inflammatory markers of health.

## 1. Introduction

Latinos who are 65 years or older are projected to increase from 2 million in 2003 to 15 million in 2050 and will compose the largest racial-ethnic group of older Americans second only to non-Latino Whites [1]. They have a disproportionately high prevalence of obesity and diabetes [2] and an increased risk of disability and frailty [3, 4]. Increasing participation in physical activity and preserving physical function in this rapidly growing minority population would reduce rates of disability [5].

Randomized clinical trials and intervention studies in older adults show that physical activity improves physical performance measures [5–9]. Biological markers have been postulated as a possible mechanism for this well-described relationship where physical activity improves measures of biological markers of health which in turn are associated with better physical performance measures [10–15] and health outcomes [3, 16–18]. Although studies have found independent associations between physical activity, physical performance, and biological markers of health in cohorts with few older adults, few of these studies focus on older minority adults [19–21]. Studies of biological markers of health may not be generalizable to community dwelling older minorities [21]. Community-level and social factors such as socioeconomic status and race ethnicity may contribute to variation in biological marker levels and health outcomes for older minorities [22–26]. We need more studies that focus on older Latinos—a growing population with a high chronic disease burden [15, 19–21, 27, 28]. Factors such as acculturation and neighborhood walkability may impact participation in physical activity among Latinos.

The present study examined the cross-sectional and longitudinal relationships between physical activity levels, physical performance, and biological markers of health among a unique sample of older sedentary Latinos with high levels of morbidity. It was hypothesized that increased physical activity would be associated with better biological markers of health, better biological markers would be associated with higher physical performance measures, and more physical activity would be associated with better physical performance. The authors were also interested in whether biological markers of health would mediate the hypothesized association between physical activity and physical performance.

## 2. Methods

### 2.1. Setting

The study was conducted using principles of community-partnered participatory research [29]. The community partners were the City of Los Angeles Department of Aging and a community advisory board consisting of six community leaders in aging. They contributed to the study design and operations—most significantly recruitment, enrollment, and selection of outcomes. One community partner (LT) participated in the project from design to dissemination of results. The community advisory board members remain very active and participate in the National Institute of Aging (NIA) funded UCLA Center for Minority Aging Research/Center for Health Improvement of Minority Elderly.

### 2.2. Study Sample

The authors analyzed baseline and 6-month follow-up data from 120 Latinos (>60 years) who participated in a supplemental study (*Caminemos Dos*) of a National Institute of Aging (NIA) funded community-based randomized clinical trial of an interdisciplinary behavioral intervention to increase walking among sedentary older Latinos (Clinicaltrials.gov Identifier #NCT00183014). Participants were recruited and enrolled in 2006-2007 from Los Angeles area senior centers. The* Caminemos* inclusion criteria were (1) age ≥ 60 years; (2) being Latino; (3) being not already engaged in ≥20 minutes of moderate intensity (or greater) physical activity ≥3 days a week; (4) no medical contraindication to participation in a physical activity class; (5) ability to participate in a 1-hour discussion session; and (6) ability to walk. Once enrolled in* Caminemos*, participants were offered the opportunity to enroll in this supplemental biological marker study (*Caminemos Dos*). Participants in the supplemental study agreed to have their blood drawn twice—once at baseline (prior to randomization) and once 6 months later. The study invited a consecutive sample of participants between June and November of 2006 and eighty percent of those invited enrolled. Participant characteristics (age, gender, education, birthplace, and mean number of medical conditions) were similar to those of the overall parent clinical trial population. The parent study is ongoing.

### 2.3. Data Collection

All data collection took place at the senior centers by trained bilingual staff and consisted of an in-person survey (interviewer-administered), brief physical examination, physical performance measures, and phlebotomy. All staff involved in data collection was blinded to whether or not subjects were in the intervention or control arm of the parent study.

### 2.4. Measures

#### 2.4.1. Biological Measurements

Fasting blood samples were collected by a licensed phlebotomist and transported directly to the UCLA Clinical Immunology Research Laboratory (CIRL) for processing and storage at −80°C until analysis. C-reactive protein (CRP) and insulin testing was performed by the CIRL. Glucose, triglycerides, and high density lipoprotein- (HDL-) cholesterol testing was performed by the UCLA Clinical Laboratory.

Serum CRP was measured by a high-sensitivity sandwich enzyme immunoassay from Immundiagnostik. Interassay precision (expressed as percent coefficient of variation) ranged from 8.1% to 11.4% over a wide range of CRP concentrations.* Plasma glucose* was measured by the glucose oxidase method (measures oxygen consumption using an oxygen detecting electrode) on a Beckman Synchron LX20 automated system.* Plasma insulin *was measured by a sandwich immunoassay on the Siemens IMMUNLITE 2000 automated system. Serum* triglycerides *were measured on the Beckman Synchron LX20 automated system by a coupled enzymatic method that produces a red-colored complex. HDL-cholesterol was quantitated on the Beckman Synchron LX20 automated system using an elimination enzymatic assay from Polymedco. All tests were performed using the same lot of reagents or assay plates in order to minimize variability due to differences in reagent lots.

#### 2.4.2. Physical Activity

Physical activity was measured using a culturally tailored modified version of the Yale physical activity survey (YPAS) that assesses time spent in twenty-nine activities in different categories (work, yard work, care taking, exercise, and recreational activities) and the intensity of activity (light, medium, or vigorous) [31]. Pennathur et al. found that the YPAS had moderate to good reliability for physical activity assessment in older Mexican-American adults [31]. The YPAS is designed for older adults and provides three indices for a typical week during the last month. A total time summary index (hours·week^−1^) was computed by summing the time spent in each activity, and an energy expenditure summary index (kcal·week^−1^) was calculated by multiplying time spent in each activity by an intensity factor and then summing across all activities. The activity dimensions summary index (total units) was computed by estimating the number of hours spent in five physical activity dimensions (vigorous activity, leisurely walking, moving, standing, and sitting), multiplying it by frequency, and multiplying it again by a weighing factor. Average steps per week at baseline were recorded using The Digiwalker (Yamax DW-500, New Lifestyles, Inc., Kansas City, MO) pedometers [32]. A description of the pedometer protocol is published elsewhere [33].

#### 2.4.3. Physical Measurements

Physical performance was measured with a short physical performance NIA battery (SPPB) that included components of gait speed, chair stands, and a balance test [34]. Each individual component was timed and given a 0–4 score (4 = best). A total performance score was calculated by summing the individual component scores into a 0–12 summary score (12 = best).* Waist circumference, height*, and* weight* were measured using a standardized protocol. Twenty percent of subjects had these measurements taken twice on the same visit in order to calculate reliability coefficients (interrater reliability 0.99; *P* < 0.001).

#### 2.4.4. Other Measures

Cognitive function was measured using the modified minimental examination (3 MS) and depressive symptoms were measured using the five-item geriatric depression scale (GDS) [35]. The GDS was dichotomized at less than 2 versus 2 or higher because of this cut point's strong independent association with clinical depression (sensitivity of 97% and specificity of 85%) [35]. Because of the distribution and sample size considerations, disability was dichotomized (any disability versus none) if a participant self-reported at least one limitation in any activities of daily living (ADLs) summary scale. Also sociodemographic and clinical characteristics were measured including age, gender, marital status, education, health status, income, birthplace, years living in the US, the presence of the metabolic syndrome [36], Charlson comorbidity index [37], and the number of hospitalizations in the past 6 months. Two items from the Behavioral Risk Factor Surveillance System Survey were used to measure smoking. Acculturation and perception of neighborhood were measured using the Marin Short Acculturation Scale and the Neighborhood Environment and Walkability Scale, respectively.

### 2.5. Statistical Analyses

Descriptive statistics were computed to summarize the baseline information and characterize the distributions of each biological marker of health. Scatter plots were generated to examine the association between each physical activity measure and six biological markers at baseline. Variable log-transformations of the outcomes were explored when normality assumption and robust inference were imperative. Pearson's correlation was used to evaluate the bivariate associations.

A series of bivariate analyses were conducted to examine whether patient sociodemographic characteristics, acculturation, neighborhood walkability, and clinical measures such as disability, cognitive function, and depressive symptoms could be confounding variables that needed to be accounted for in regressions. Baseline cross-sectional, multivariable regression analyses were performed to estimate the association between physical activity levels (YPAS indices and pedometer step count) and each physical performance measure. Physical performance measures at the six-month followup were not collected. For the longitudinal analyses, change in physical activity, as measured with the YPAS indices, was individually regressed one by one against change in each of the biological markers of health. For both cross-sectional and longitudinal regression analyses, a first set of models, “model 1,” controlled for age and gender. The second set of models, “model 2,” controlled for model 1 covariates, tobacco smoking, Charlson comorbidity score, and disability. In separate sensitivity analyses, the regressions controlled for body mass index [28], history of recent hospitalization, senior center site, perceived neighborhood walkability, acculturation, and study arm (corresponding to the parent clinical trial) but the results did not appreciably change the findings. In the interest of parsimony and given the sample size considerations, the final regression models did not include these covariates. *P* value of <0.05 was considered statistically significant for all analyses. Stata version 11 (Stata Corp., College station, Texas) statistical software was used to conduct all analyses.

To examine if biomarkers of health were mediators, biomarkers were first regressed on physical activity, then physical performance on physical activity, and finally physical performance on both physical activity and biomarkers [38].

## 3. Results

The majority of the sample was female, completed eight or less years of education, and self-reported poor/fair health status. About one in five participants had a yearly income of $20,000 or greater. The majority of participants were born in Mexico. More than half had 3 or more selected chronic conditions and 16% had been hospitalized in the last six months (Table 1).

Table 2 includes descriptive results for the biological markers measured plus metabolic syndrome. Almost half of the sample had a CRP >3 mg/dL and a fasting glucose level >100 mg/dL (Table 2). More than half of the participants had elevated blood pressure (systolic blood pressure ≥ 140 mmHg or diastolic blood pressure ≥ 90 mmHg), and almost half had their triglycerides ≥150 mg/dL.

Participants with metabolic syndrome had a higher mean CRP (5.3 versus 4.4 mg/dL; *P* = 0.45), insulin (18 versus 11 uIU/mL; *P* = 0.07), and BMI (32 versus 30 kg/m^2^; *P* = 0.45) compared to their counterparts. There were no significant differences in 3 MS, disability, or GDS among those with metabolic syndrome compared to their counterparts. Biological markers did not correlate with 3 MS or GDS scores (data is not shown), but those with any disability had higher mean CRP (6.40 versus 4.30 mg/dL), insulin (14.8 versus 11.3 uIU/mL), and waist circumference (113 versus 105 cm) measures (all *P* < 0.05). Those without any disability had higher mean baseline physical performance scores (8.4 versus 7.2; *P* = 0.003) and seven-day average pedometer step counts (2916 versus 2263; *P* = 0.03). GDS and 3 MS scores did not vary by physical performance or physical activity levels as measured. Perception of neighborhood walkability and acculturation were not associated with biological markers of health, physical activity, or physical performance.

Table 3 includes findings for the unadjusted and adjusted cross-sectional analyses of baseline data that examines the relationship between physical activity measures and physical performance outcomes. In final adjusted models, greater energy expenditure and total time YPAS indices were significantly associated with faster gait speed.

In unadjusted longitudinal change-change analyses (Table 4), change in YPAS total time index correlated inversely with change in insulin (beta coefficient [*β*] = −0.022, *P* = 0.02). After adjusting for covariates in models 1 and 2, this correlation was not significant. After adjusting for covariates in the final regression models, change in physical activity level (YPAS activity index) correlated inversely with change in CRP (*β* = −0.051; *P* = 0.03). Because biomarkers were not significantly associated with physical performance, the conditions outlined by Baron and Kenny [38] for a mediation analysis did not hold and therefore the likelihood that biomarkers mediated the effect of physical activity on physical performance was low.

This study served as a stimulus for a successful grant submission to the UCLA Clinical and Translational Science Institute to study effective community-partnered approaches to recruit and retain older African-American and Latinos in studies that include the measurement of biological markers of health. Many of the academic and community partners collaborated to provide infrastructure to recruit minority seniors through the NIA funded Los Angeles Community Academic Partnership for Research in Aging (L. A. CAPRA).

## 4. Discussion

This community-partnered study of urban sedentary older Latinos found that positive changes in physical activity levels were independently associated with negative change in CRP and waist circumference. As expected, higher physical activity levels correlated positively with higher physical performance. While these associations have been described previously in nonminority samples, these new findings in this difficult-to-reach urban sample of older sedentary Latinos strengthen the public health imperative to increase physical activity among older adults. Because of the unprecedented growth of Latino populations in many communities across the US and the disproportionate levels of diabetes, metabolic syndrome, and hypertension among older Latinos, these findings point to a great need to examine these associations further in future, larger prospective studies of older Latinos. Levels of biomarkers for this population are higher than that reported in national samples [39]. This could be because this unique sample is sedentary, low SES, and from an urban community [40]. This sample had a higher prevalence of diabetes and lower educational levels compared to populations from other studies [5, 11–13].

The finding that self-reported physical activity was inversely and independently associated with CRP is similar to other studies in non-Latino populations [11, 13, 41]. The significant cross-sectional association between higher self-reported physical activity levels and better physical performance is likewise in agreement with other studies [11–13]. Though other studies have found a significant positive association between biomarkers and physical performance, the current study did not and therefore the measured biomarkers were likely not mediators for the significant relationship between physical activity and physical performance [38]. Possible reasons for this finding are the limited sample size and the possibility that the SPPB was not highly predictive of levels of biomarkers in community dwelling older Latinos as previously reported in other similar populations [41].

A strength of this study is that the population reflects a “real world” setting by including older Latinos with multiple chronic conditions from the community who are difficult to recruit, enroll, and retain in health studies and therefore are underrepresented in existing databases in which the association between physical activity and biological markers of health has been examined [11, 13]. Community-partnered research methods were critical for the recruitment of this hard-to-reach population. This study also had several limitations. The generalizability is limited as the sample was a convenience sample from senior centers of an ongoing clinical trial and participants were mainly Mexican-American females. Because the sample size is small, the data could not be stratified by gender. Six months was likely not that enough time to fully capture changes in some biological marker levels because multiple data points across time were not available for the analyses. The study aims were hypotheses generating and as such did not set out to disprove a null hypothesis. Data on the use of anti-inflammatory medication that may influence levels of biomarkers was not available [11, 18]. The survey measures (YPAS) were self-reported and could be affected by recall bias. However, the YPAS has been validated in Spanish [31, 42, 43].

This study has research implications. Investigators need to recruit minority seniors into clinical trials and intervention studies that investigate the biological pathways between health behaviors such as physical activity and clinical outcomes. Minorities are underrepresented in aging studies despite having a disproportionately high burden of disease from chronic conditions.

In summary, in these exploratory analyses, physical activity levels were inversely associated with some biological markers of health and positively associated with physical performance measures among older Latinos with a high prevalence of chronic conditions. Studies continue to support the potentially high impact of effective physical activity interventions in urban minority communities.

## Figures and Tables

**Table 1:** Characteristics of participants (*n* = 120).

Characteristic	*n* (%)
Age (years)	
60–69	41 (34)
70–79	57 (48)
80+	22 (18)
Female	96 (80)
Marital status	
Never married	13 (11)
Married	26 (22)
Separated/divorced	28 (23)
Widowed	53 (44)
Education	
No schooling	16 (13)
≤8th grade	45 (38)
≥9th grade (some high school or more)	59 (49)
Health status	
Excellent/very good	14 (12)
Good	37 (39)
Fair/poor	69 (57)
Income	
<$7,500	21 (21)
$7,500–<$10,000	25 (24)
$10,000–<$12,500	13 (13)
$12,500–<$15,000	8 (8)
$15,000–<$20,000	12 (12)
$20,000+	24 (24)
Birthplace	
United States	7 (7)
Mexico	77 (81)
Other	11 (12)
Length of US residence (years)	
1–30	15 (20)
31–40	20 (27)
41–50	20 (27)
50+	20 (27)
Lifetime smoking, cigarettes	
<100	80 (67)
≥100	40 (33)
Body mass index (kg/m^2^)	
Healthy (18.6–24.9)	17 (14)
Overweight (25–29.9)	38 (32)
Obese (≥30)	64 (54)
Diabetes, *n* (%)	48 (40)
Medical conditions ≥3	65 (54)
Hospitalized in past 6 mo	19 (16)
5-item geriatric depression scale score ≥2 (reference <2)	29 (24)
Modified minimental state (3MS) ≥80	78 (73)
Ever have ADL impairment (reference never have)	41 (34)

**Table 2:** Descriptive profiles for biological markers of health for participants (*n* = 120).

Biological markers	Mean (SD)	Median	IQR* range	% Above clinical cut points^†^
Inflammatory				
C-reactive protein (mg/L)	5.03 (5.77)	2.73	1.59, 5.64	46
Metabolic				
Glucose (mg/dL)	114 (45)	98	91, 126	48
Insulin (uIU/mL)	16 (20)	12	7, 18	
Triglycerides (mg/dL)	166 (85)	148	110, 189	47
HDL-cholesterol (mg/dL)	50 (14)	48	42, 56	55
Body mass index (kg/m^2^)	31 (7)	30	27, 35	53
Waist circumference (cm)	108 (15)	107	98, 118	83
Physiologic				
Systolic blood pressure (mm Hg)	142 (20)	141	131, 152	53
Diastolic blood pressure (mm Hg)	74 (11)	74	66, 81	7
Metabolic syndrome, ATPIII criteria (range 0–5)^‡^	3.1 (1.2)	3.0	2.0, 4.0	68

SD = standard deviation.

*IQR = interquartile range.

^†^Clinical cut points: CRP > 3 mg/dL; glucose ≥ 100 mg/dL; triglycerides ≥ 150 mg/dL; HDL <40 (men) or <50 (women); waist circumference >102 cm (men) or >88 (women); BMI ≥ 30 kg/m^2^; systolic blood pressure ≥ 140 mm Hg; and diastolic blood pressure ≥ 90 mm Hg.

^‡^Meets 3/5 ATP III criteria for metabolic syndrome (source: [30] ). Five criteria include (1) glucose ≥ 100 mg/dL; (2) triglycerides ≥ 150 mg/dL; (3) HDL < 40 (men) or <50 (women); (4) waist circumference > 102 cm (men) or >88 cm (women); and (5) blood pressure ≥ 135/85 mm Hg.

**Table 3:** Cross-sectional unadjusted and adjusted linear regression models of physical performance as a function of physical activity measures among participants (*n* = 120).

Yale physical activity indices^§^ and seven-day average pedometer step count	Physical performance^‡^			
	Gait speed	Chair stands	Balance test	Total score
	*β*-coef	*β*-coef	*β*-coef	*β*-coef
Total time index (hours*·*week^−1^)				
Unadjusted	.034^†^	.024	.008	.065*
Model 1	.035^†^	.019	.009	.064*
Model 2	.026*	.010	.005	.040
Activity summary index (total units ∗ 100)				
Unadjusted	.100	.953	.536	1.67
Model 1	.028	.982	.531	1.54
Model 2	.311	.726	.393	.828
Energy expenditure index (kcal*·*week^−1^ [100])				
Unadjusted	.015^†^	.011*	.004	.030^†^
Model 1	.015^†^	.010	.004	.029*
Model 2	.010*	.005	.002	.017
Pedometer step count (average/week ∗ 100)				
Unadjusted	.010	.012	.010*	.030*
Model 1	.010	.010	.010*	.298*
Model 2	.004	.006	.008	.018

*P value < 0.05.

^†^ *P* value < 0.01.

^‡^Short physical performance NIA battery.

^§^Measured with Yale physical activity survey.

*Notes*. (1) Model 1 controlled for age and gender. (2) Model 2 controlled for Model 1 covariates, tobacco use, Charlson comorbidity index, and disability.

**Table 4:** Longitudinal (6 months) unadjusted and adjusted linear regression models of change in biological markers as a function of change in physical activity among participants (*n* = 120).

Physical activity indices^‡^	CRP (mg/dL)	Insulin (uIU/mL)	HDL-cholesterol (mg/dL)	Glucose (mg/dL)	Triglycerides (mg/dL)	Waist circumference (cm)
	*β*-coef	*β*-coef	*β*-coef	*β*-coef	*β*-coef	*β*-coef
Total time index (hours*·*week^−1^)						
Unadjusted	−.055	−.022*	.023	.168	−.062	−.026
Model 1	−.051	−.029	.033	.185	−.164	−.013
Model 2	−.057	−.057	.033	.296	−.103	−.007
Activity index (total units)						
Unadjusted	−.045*	−.070	−.027	.099	.356	−.168^†^
Model 1	−.052*	−.070	−.017	.074	.373	−.167^†^
Model 2	−.051*	−.052	−.022	.137	.437	−.164*
Energy index (kcal*·*week^−1^ [100])						
Unadjusted	−.002	−.005	−.005	.059	.092	−.023
Model 1	−.001	−.009	−.001	.082	.014	−.016
Model 2	−.002	−.021	.002	.145	.044	−.017

*P value < 0.05.

^†^ *P* value < 0.01.

^‡^Measured with Yale physical activity survey.

CRP = C-reactive protein.

HDL = high density lipoprotein.

*Notes*. (1) Model 1 controlled for age and gender and (2) model 2 controlled for model 1 covariates, tobacco use, Charlson comorbidity index, and disability.
